# Zika and Chikungunya virus co-infection in a traveller returning from Colombia, 2016: virus isolation and genetic analysis

**DOI:** 10.1099/jmmcr.0.005072

**Published:** 2016-12-19

**Authors:** Kartikeya Cherabuddi, Nicole M. Iovine, Kairav Shah, Sarah K. White, Taylor Paisie, Marco Salemi, J. Glenn Morris Jr, John A. Lednicky

**Affiliations:** ^1^​ Division of Infectious Diseases and Global Health, Department of Medicine, College of Medicine, University of Florida, Gainesville, FL, USA; ^2^​ Emerging Pathogens Institute, University of Florida, Gainesville, FL, USA; ^3^​ Department of Environmental and Global Health, College of Public Health and Health Professions, University of Florida, Gainesville, FL, USA; ^4^​ Department of Pathology, Immunology, and Laboratory Medicine, College of Medicine, University of Florida, Gainesville, FL, USA

**Keywords:** Zika, Chikungunya, Arthralgia, co-infection, phylogenetic analysis, virus isolation

## Abstract

**Introduction:**

*Zika* *virus* (ZIKV) and *Chikungunya* *virus* (CHIKV) can share the same mosquito vector, and co-infections by these viruses can occur in humans. While infections with these viruses share commonalities, CHIKV is unique in causing arthritis and arthralgias that may persist for a year or more. These infections are commonly diagnosed by RT–PCR-based methods during the acute phase of infection. Even with the high specificity and sensitivity characteristic of PCR, false negatives can occur, highlighting the need for additional diagnostic methods for confirmation.

**Case presentation:**

On her return to the USA, a traveller to Colombia, South America developed an illness consistent with Zika, Chikungunya and/or Dengue. RT-PCR of her samples was positive only for ZIKV. However, arthralgias persisted for months, raising concerns about co-infection with CHIKV or *Mayaro* viruses. Cell cultures inoculated with her original clinical samples demonstrated two types of cytopathic effects, and both ZIKV and CHIKV were identified in the supernatants. On phylogenetic analyses, both viruses were found to be related to strains found in Colombia.

**Conclusion:**

These findings highlight the need to consider CHIKV co-infection in patients with prolonged rheumatological symptoms after diagnosis with ZIKV, and the usefulness of cell culture as an amplification step for low-viremia blood and other samples.

## Introduction

The arboviruses *Zika virus* (ZIKV; Family *Flaviviridae,* Genus *Flavivirus*) and *Chikungunya virus* (CHIKV; Family *Flaviviridae*, Genus *Alphavirus*) are RNA viruses that are most commonly transmitted by mosquitos, including *Aedes spp*. In Colombia, cases of CHIKV infection were first identified in July 2014 (Cardona-Ospina *et al.*, 2015) followed by cases of ZIKV infection in October 2015 (Pacheco *et al.*, 2016). Both viruses cause similar initial symptoms with some distinctions, but a key difference between them is the short time course of ZIKV infection of two weeks or less, whereas infection with CHIKV can lead to arthritis and arthralgias that may persist for a year or more. Since the clinical signs of arboviral infection are not unique for specific viruses, RT-PCR-based methods are preferred for making diagnoses during the acute phase of infection. However, despite PCR’s high sensitivity, false negatives may occur in specimens with low levels of virus. Therefore, inoculation of suspected arbovirus-containing human samples onto cell cultures may allow for replication of virus, which can then be detected by RT-PCR. Here we describe the clinical presentation and course of a patient with dual ZIKV and CHIKV infection diagnosed by a combination of RT-PCR performed directly on the patient’s clinical specimens as well as on spent cell culture media.

## Case report

A 40-year-old woman and her husband had recently travelled from the United States to Bogota, Colombia for 7 days. They spent time outdoors in both urban and rural areas. She recollects having had mosquito bites and had three bite marks on her leg. She was asymptomatic during her stay.

**Fig. 1.**
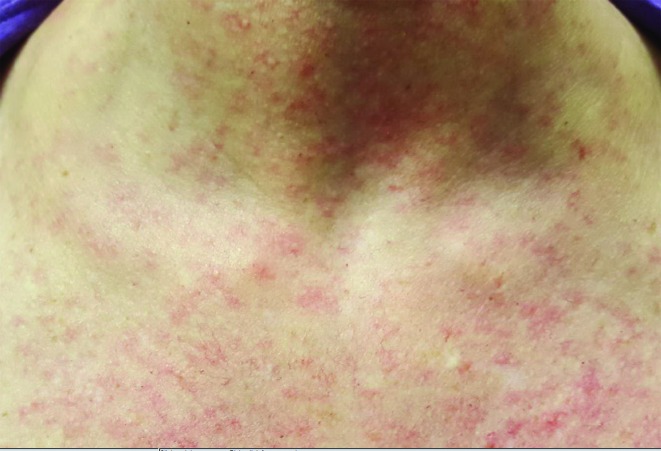
Maculopapular rash over neck and trunk.

On day 3 after returning to the USA, she developed itching of her scalp. On day 4, she felt fatigued and developed low-grade fever and back pain. On day 5, she presented to the outpatient infectious diseases clinic for evaluation after her scalp became erythematous and she started developing a pruritic, maculopapular rash on her face and trunk (Fig. 1) that rapidly spread over her entire body. Her wrist and ankle joints became very painful and swollen. She also developed a pressure-like sensation behind her eyes with conjunctival redness.

She had received yellow fever vaccine previously. She lived in an area of Florida with no local transmission of CHIKV or DENV. There had been no other recent travel. Her husband who travelled with her was asymptomatic and was not tested.

## Investigations

On day 5, laboratory studies were performed and revealed a white cell count of 2800 mm^−3^ (normal range: 4000–10 000 mm^−3^) with 56 % neutrophils, 38 % lymphocytes and 1.1 % eosinophils (normal range: 40–80 %, 20–40 % and 0–8 % respectively). Haemoglobin and platelet counts were normal. Serum electrolytes, creatinine and hepatic aminotransferases were normal. Urine pregnancy test was negative.

**Fig. 2.**
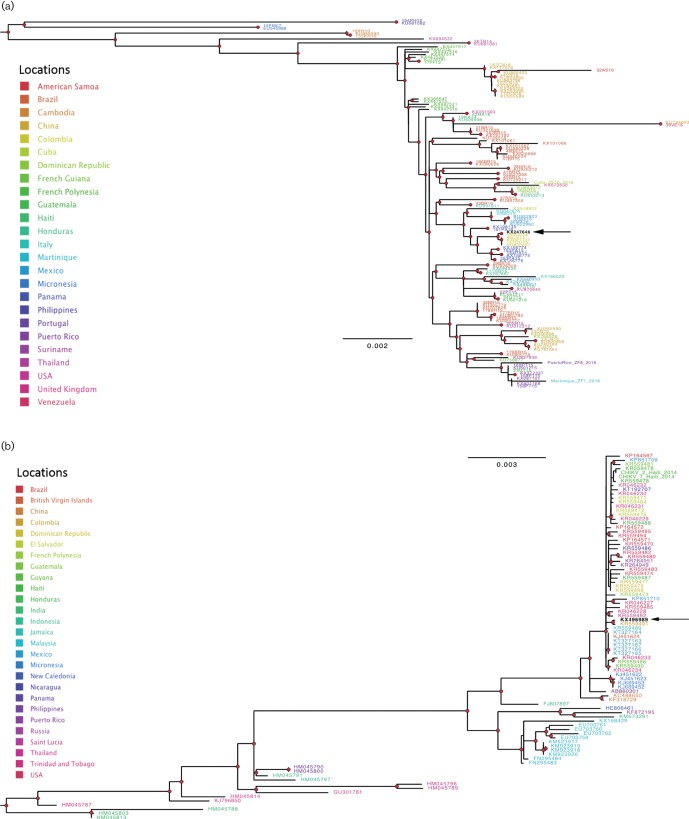
Maximum-likelihood phylogenies of ZIKV and CHIKV strains. The ML trees were inferred using all currently available full-genome sequences with the best nucleotide substitution model and the ML algorithm implemented in the program IQ-TREE (Nguyen *et al.*, 2015). Trees were mid-point rooted. Branch lengths are drawn proportionally to nucleotide substitutions per site as indicated by the bar at the bottom of each tree. Tips were labeled according to the GenBank accession number of each sequence and coloured to indicate the country of origin according to the legends in the figure. Red circles indicate strong bootstrap support (>90% in 1000 bootstrap replicates) for the subtending clade. Arrows indicate the ZIKV and CHIKV strains sequenced in the present study. (a) ZIKV phylogeny. For display purposes only the South American clade is shown, the full tree is given in Fig. S1a. (b) CHIKV phylogeny. For display purposes only the major clade including the new isolate is shown, the full tree is given in Fig. S1b.

Saliva, serum and urine samples were sent to the Florida State Laboratory as she fulfilled criteria for ZIKV testing. Reverse transcription–polymerase chain reaction (RT–PCR) tests for the viral genomic RNAs (vRNAs) of ZIKV, CHIKV and *Dengue virus* (DENV) 1,2,3,4, and ELISA tests for ZIKV, CHIKV and DENV IgM antibodies were performed, as well as an IgG assay for DENV. All the tests from the Florida State Lab were negative, with the exception of a positive RT-PCR assay for ZIKV vRNA. Saliva, serum and urine samples were also sent to the research laboratories at the University of Florida Emerging Pathogens Institute, Gainesville, where aliquots of each were inoculated onto cell cultures for virus isolation, and tested by RT-PCR. Each sample was positive for ZIKV vRNA by RT–PCR but negative for DENV and CHIKV vRNAs. ZIKV was isolated from each of the saliva, serum and urine samples (verified by RT–PCR); perinuclear vacuoles characteristic of ZIKV (Iovine *et al.*, 2016; Lednicky *et al.*, 2016b) were first evident in LLC-MK2 cells 72 h post-infection (data not shown). As described in the online Supplementary Materials and Methods, ZIKV vRNA from virions in the spent cell growth media was detected by RT–PCR using primers ZIKVF9027–ZIKVR9197c (Balm *et al.*, 2012) and 9271–9373 (Faye *et al.*, 2013). The complete nucleotide sequence of the ZIKV genome was determined by using a genome-walking strategy, as previously described (Lednicky *et al.*, 2016b). The maximum-likelihood (ML) tree, inferred from all available full genome sequences downloaded from GenBank as previously described (Lednicky *et al.*, 2016b), showed that the ZIKV sequence clustered within a highly supported clade (bootstrap>90 %) including sequences isolated from patients in Colombia (Figs 2a and S1a, available in the online Supplementary Material).

## Diagnosis

As discussed above, the patient's saliva, serum and urine were positive for ZIKV and she was asked to follow general precautions and abstain from sexual activity. On day 9, she continued to have severe fatigue, worsening joint pain and swelling and since the DENV RT-PCR was negative she was started on ibuprofen. Rash was significantly better but persisted on the torso and legs for another week. On day 16, she returned to work, though fatigue and joint discomfort persisted and she did not feel well enough to exercise.

Two months after the initial infection, she continued to experience severe arthralgias involving her wrists bilaterally and the plantar surface of the left foot. She was seen in the orthopedic clinic 3 months after initial illness and noted to have tenderness of the second and third left metatarsal heads. Plain radiographs of the left foot revealed no fractures or soft-tissue swelling. It was recommended that she wear a brace for 3 weeks.

**Fig. 3.**
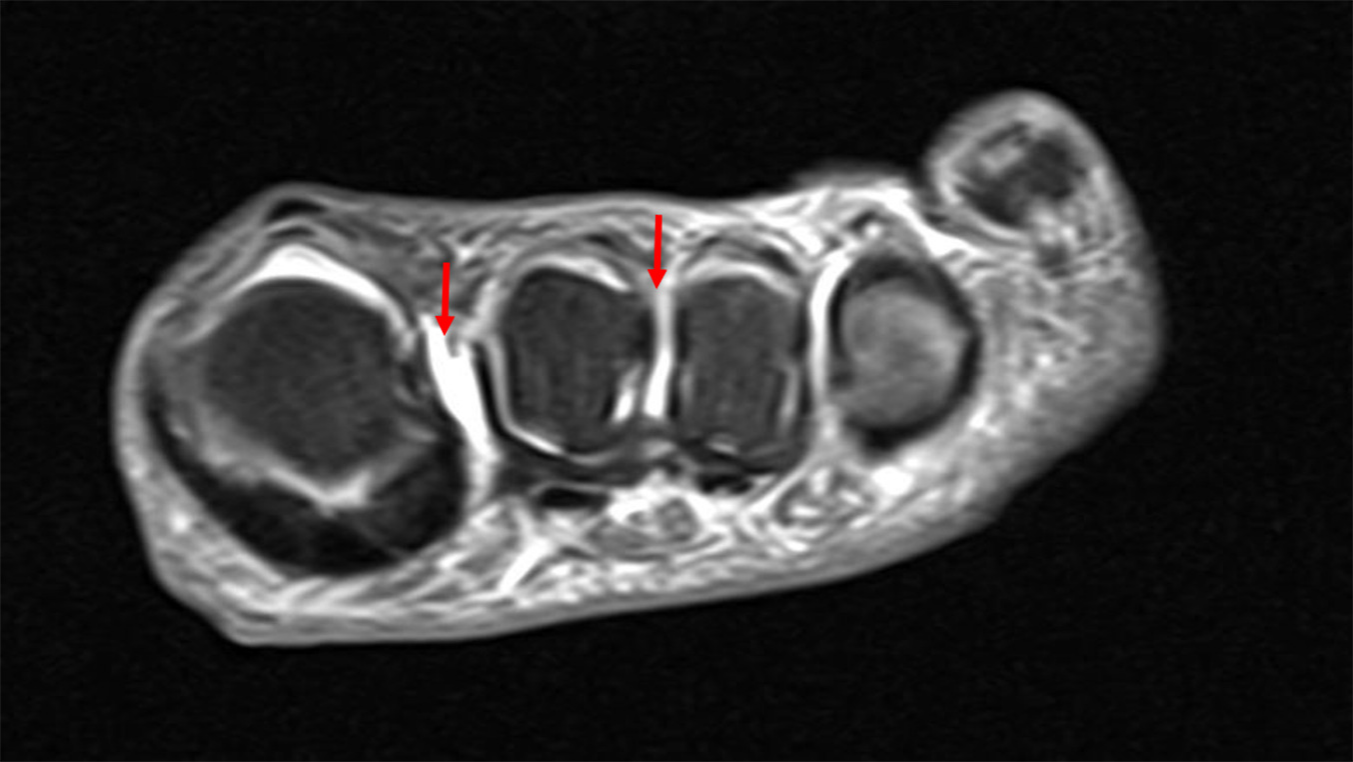
T-2 weighted magnetic resolution imaging of the left foot without contrast showing trace fluid in the intermetatarsal bursae between the 1st and 2nd metatarsal heads and 2nd and 3rd metatarsal heads (red arrows).

**Fig. 4.**
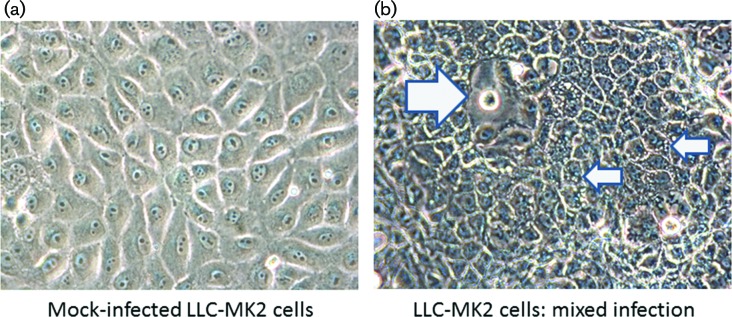
Mixed virus CPE revealed by plaque assay. (a) Non-infected LLC-MK2 cells. (b) Virus-infected cells; the cells are darker, and ZIKV-infected cells (small arrows) are vacuolated, whereas a plaque formed by CHIKV is easily distinguishable (large arrow). Original images taken at a magnification of 400X.

Five months after illness onset, she continued to have persistent pain and a magnetic resonance imaging study of the left foot showed trace fluid in the intermetatarsal bursae between the first and second metatarsal heads and second and third metatarsal heads (Fig. 3). Her symptoms prompted a re-evaluation of the viral isolation studies, which were initially terminated 9 days’ post-inoculation upon isolation of ZIKV. A frozen aliquot of the first-passage virus preparation was thus thawed and inoculated onto Vero and LLC-MK2 cells to determine whether another virus(es) was present but had been overlooked. It was noted that a second virus was present that displayed cytopathic effects (CPE) more consistent with findings expected for alphaviruses: lytic infection/apoptosis of infected Vero and LLC-MK2 cells and, as observed by others, cellular blebs (Krejbich-Trotot *et al.*, 2011; Wikan *et al.*, 2012) were seen. Under an agarose overlay used to purify the two different viruses, clear plaques formed by the second virus were discernible even among ZIKV-infected cells (Fig. 4). As described in the Supplementary Materials and Methods, CHIKV vRNA was detected in spent-cell culture media by using the CDC Real Time RT-PCR for Detection of Chikungunya Virus (version 26 June 2014). The complete nucleotide sequence of the CHIKV genome was determined using a genome-walking strategy (Supplemental Materials and Methods), with the primers reported in Table S1. Similar to the ZIKV sequence, the ML tree, inferred from full-genome reference sequences downloaded from GenBank, clustered the CHIKV sequence with another CHIKV strain previously isolated in Colombia with strong support (bootstrap>90 %) (Figs 2b and S1b).

## Discussion

While over 80 % of ZIKV infections in adults are thought to be asymptomatic (Duffy *et al.*, 2009), common manifestations include mild fever, rash and conjunctivitis, with headache, retro-orbital pain and myalgias reported less commonly (CDC, 2016). In pregnant women infected with ZIKV, particularly in the first trimester, serious adverse fetal outcomes associated with brain/neurological development are well recognized. Patients with ZIKV infections also appear to have an increased risk of post-infectious neurological complications such as Guillian–Barre syndrome (Wikan & Smith, 2016). Arthritis and arthralgias have been described at a varying rate ranging from 14% to 65 % in previous outbreaks in Indonesia, Micronesia, French Polynesia and Brazil (Brasil *et al.*, 2016; Duffy *et al.*, 2009; Fernanda Estofolete *et al.*, 2016; Wikan & Smith, 2016), however none of these case series have reported longer-term rheumatological sequelae. In Micronesia, the median duration of arthralgia was 3.5 days with a range of 1–14 days (Duffy *et al.*, 2009). While the basic constellation of symptoms including fever and rash is common to many arboviruses, including ZIKV, CHIKV and DENV, there are some differences in symptomatology: in particular, conjunctivitis has been linked more commonly with ZIKV, with severe debilitating arthralgias occurring more commonly with DENV and CHIKV (CDC, 2016), and more prolonged arthralgias and rheumatological symptoms occurring with CHIKV. In this particular case, the occurrence of persistent arthralgias in a patient with ZIKV suggested the possibility of a dual infection, with either CHIKV or *Mayaro virus* (MAYV), as was subsequently confirmed in the laboratory.

Coinfections with ZIKV and DENV, and DENV with MAYV with virological confirmation are well described (Iovine *et al.*, 2016; Lednicky *et al.*, 2016b). Coinfection of ZIKV with CHIKV (Sardi *et al.*, 2016; Zambrano *et al.*, 2016) and ZIKV and CHIKV with DENV have also been reported (Villamil-Gomez *et al.*, 2016). Our studies highlight the importance of not assuming that an initial report of a positive RT-PCR for one arboviral agent such as ZIKV excludes the possibility of infection with other agents, which might have been transmitted by the same mosquito or other mosquitos in the same area. In this case, we propose that the initial negative RT-PCR for CHIKV reflected a low viral titre early in the incubation period that was simply not detected by the assay. Failure to identify IgM antibodies to ZIKV and CHIKV is not unexpected early in the course of the infection. The other hypothesis for low viremia with CHIKV is if one virus could suppress another *in vitro*. Whereas one of the authors (J. L.) has experienced/observed the effects *in vitro*, there are, to our knowledge, no published studies (by J. L. or others) that specifically document suppression/interference by alphavirus and flavivirus co-infections of susceptible cells *in vitro*. A plausible explanation is that the cellular pathways for alphavirus and flavivirus maturation are different, so interference is possible. In a mixed infection by an alphavirus and a flavivirus *in vitro*, the ‘winner’ depends upon which virus dominates first, which, in turn, depends on the viral growth kinetics in a particular type of cell, and the original multiplicity of infection of each virus.

However, on viral culture it was possible to clearly demonstrate the presence of CHIKV, in keeping with the patient’s clinical presentation. The sequence data provide further confirmation that the patient was infected with both ZIKV and CHIKV. The phylogenetic analysis clearly demonstrated that both strains were closely related to strains circulating in Colombia, thus indicating that both infections were probably acquired during the trip to the South American country. Since we had no other arboviral strains in the laboratory from Colombia, it is very unlikely that the phylogenetic findings were the result of laboratory cross-contamination.

In conclusion, prolonged symptoms of arthritis or arthralgias in patients with ZIKV infection should prompt evaluation for other arboviruses such as CHIKV and, possibly, MAYV, another alphavirus similar to CHIKV that our group has recently identified in Haiti (Lednicky *et al.*, 2016a). Current diagnostics, including RT–PCR, are technically complex, expensive, and are not 100 % sensitive; serology also presents difficulties, particularly within the flavivirus group where there is substantial cross-reactivity among species (Calisher *et al.*, 1989). In this setting, there is an urgent need for development of alternative testing approaches, merged with a better understanding of sample collection and handling techniques, to minimize the logistical and financial burden associated with laboratory analysis in developing-world settings. In our patient’s case, it was helpful to elucidate the aetiology of her joint pain and also to understand that long-term arthralgias are inconsistent with ZIKV, which could have been contemplated with her presentation. With the expanding geographic distribution of infections with CHIK, DENV, ZIKV and now MAYV, the possibility of dual infection should always be kept in mind; at least for the present, viral culture by an experienced laboratory group would appear to provide the most sensitive means of diagnosis and assisting in determining clinical attributes and epidemiology of emerging viral infections.

## Supplementary Data

520 Supplementary File 1
