# Supplementary material for: Zika and Chikungunya virus co-infection in a traveller returning from Colombia, 2016: virus isolation and genetic analysis
Source: JMM Case Rep. 2016 Dec 19;3(6):e005072. doi: 10.1099/jmmcr.0.005072 (PMC5343122; doi:10.1099/jmmcr.0.005072)

**Zika and Chikungunya Virus Co-Infection in a Traveler Returning from Colombia, 2016**

**Supplemental Materials and Methods**

Patient specimens

Blood, saliva, and urine samples were obtained from the patient 6 days after development of symptoms. Saliva was collected by swabbing the patient’s salivary glands using a Copan flocked nylon swab (Copan Diagnostics, Inc., Murrieta, CA), which was then inserted into a Copan transport tube containing universal transport medium (UTM) and glass beads. Blood was collected into acid citrate dextrose “yellow top” tubes (ACD Vacutainer blood collection tube, Becton Dickenson and Company). Urine was aseptically collected in a 50 mL sterile polypropylene tube. The specimens were transported to the laboratory within 5 minutes of collection. The swab was swirled against the inside of the collection tube to extrude saliva, the tube vortexed for 1 minute for the glass beads to rupture cells, and the resulting suspension aliquoted, whereas urine was mixed and aliquoted without additional processing of the sample. Likewise, blood was thoroughly mixed and aliquoted without further manipulations. Aliquots were inoculated onto cell cultures and tested by RT-PCR. The remaining aliquots were cryopreserved at -80°C prior to additional analyses.

Cell culture

Mammalian cell lines LLC-MK2 (CCL-7), MRC5, (CCL-171),Vero E6 (CRL-1586), and the mosquito cell line C6/36 (CCL-75) were obtained from the American Type Culture Collection (ATCC, Manassas, VA) and propagated as described by (Iovine et al., 2016) .

Extraction and RT-PCR detection of ZIKV genomic RNA in fresh blood, saliva, and urine

Viral genomic RNA (vRNA) was extracted from virions in blood, saliva, and urine using a QIAamp Viral RNA Mini Kit (Qiagen Inc., Valencia, CA) as described previously (Iovine et al., 2016). The extracted vRNAs were subsequently analyzed by RT-PCR using primers (Santiago et al., 2013) for *Dengue virus* types 1-4, and for ZIKV vRNA using primers ZIKVF9027-ZIKVR9197c (Balm et al., 2012), 9271-9373 (Faye et al., 2013), and 835 – 911c (Lanciotti et al., 2008).

Inoculation of cell cultures

Sub-confluent cells grown in canted-neck filter-cap flasks (25 cm^2^ growing surface) and in a minimal amount of cell growth medium were inoculated with 50 µL of sample material (saliva or urine) and incubated at the appropriate temperature (28°C for C6/36 cells, mammalian cells at 35°C) for 2 hrs. Thereafter, additional cell growth medium was added, and the cells monitored daily. The cells were re-fed with maintenance medium every three days.

Detection and sequencing of ZIKV vRNA in spent cell media

Virus-specific cytopathic effects (CPE) consisting of perinuclear vacuolation and non-specific cell degeneration were observed in LLC-MK2, MRC5, and Vero E6 cells within 3 days post-inoculation with the blood, saliva, and urine samples, but were more prominent in LLC-MK2 cells at early times post-inoculation than in the other cells. In contrast, CPE were subtle in C6/36 cells up to termination of the virus isolation efforts (9 days post-inoculation). ZIKV vRNA from virions in the spent cell growth media was detected by RT-PCR using primers ZIKVF9027-ZIKVR9197c (Balm et al., 2012) and 9271-9373 (Faye et al., 2013).

Sequencing of ZIKV

The complete nucleotide sequence of the ZIKV genome was accomplished using a genome walking strategy and the methods and primers described in (Lednicky et al., 2016b). The ZIKV genomic consensus sequence of this isolate was based on vRNA purified from the spent media of LLC-MK2 cells that had been collected 7 days after they were inoculated with urine, and is designated ZIKV Homo sapiens/COL/UF-1/2016. The GenBank accession number is KX247646.1.

Detection and sequencing of CHIKV vRNA in spent cell media

To explore the possibility that another virus was initially present at low levels and over-looked, cryopreserved aliquots of passage 1 blood, saliva, and urine isolates of ZIKV from Vero E6 and LLC-MK2 cells were inoculated onto VERO E6 and LLC-MK2 cells. It was noted that aside from vacuolated cells typical for ZIKV, a second distinct type of CPE (lytic CPE) was observed in cells inoculated with the ZIKV preparations arising from blood, and an alphavirus was suspected: *West Nile, Mayaro*, and *Chikungunya viruses* were ruled in/out using RT-PCR tests (details provided upon request). *Chikungunya virus* vRNA was detected using the CDC Real Time RT-PCR for Detection of Chikungunya Virus (version 26 June 2014). The complete nucleotide sequence of the CHIKV genome was accomplished using a genome walking strategy and primers described in Table X. The 5’ end was determined by first dephosphorylating the vRNA with calf-intestinal phosphatase, decapping the dephosphorylated vRNA with tobacco acid pyrophosphatase, ligating 5’RACE roligo (Table S1) using T4 RNA ligase, then performing RT-PCR with primers 5’ RACE DNA primer and 5’ RACE R (Table S1). The 3’ end was determined using RT-PCR primers 3’ RACE F and T25G (Table S1). The CHIKV genomic consensus sequence of this isolate was based on vRNA purified from the spent media of VERO E6 cells that had been collected 5 days after they were inoculated with blood, and is designated CHIKV Homo sapiens/COL/UF-1/2016. The GenBank accession number is KX496989.1

Plaque assay

A standard agarose-overlay method was used for plaque assays. Briefly, freshly confluent LLC-MK2 and Vero E6 cells grown in six-well plates were inoculated with 1 mL serial dilutions of the virus preparations and allowed to adsorb for 1 hr at 37°C, with rocking of the plates performed at 15 min intervals. After 1 hr of incubation, the inoculum was removed, monolayer washed with serum free EMEM and replaced with 3 ml of agarose medium with 3% low IgG heat-inactivated gamma-irradiated FBS. A second overlay (1 ml) was added 3 days later containing neutral red, and the cells observed 6 days post-inoculation.

**Table S1:** CHIKV sequencing primers

| primer | sequence |
| --- | --- |
| 5’ RACE roligo | rArGrC rArUrC rGrArG rUrCrG rGrCrC rUrUrG rUrUrG rGrCrC rUrArC rUrGrG |
| 5’RACE DNA primer | AGC ATC GAG TCG GCC TTG TTG GCC TAC TGG |
| 5’ RACE R | ggcgcactacctatatccaggatgg |
| Primer 1 F | atggctgcgt gagacacacg tag |
| Primer 2 F | cccatcatgg attctgtgta c |
| Primer 1 R | gtagcatgcggctttccgggtaaagc |
| Primer 2 R | gttgaacataatcctatgttcttag |
| Primer 3 F | gacctgaca gaaggtagac gagg |
| Primer 4 F | gtgcgaccgt gtgctgttct cag |
| Primer 3 R | gcatcctgggcttcttgggcatttccg |
| Primer 4 R | ccttgcgtaacagccacttgattc |
| Primer 5 F | gtggtcgtcc gggttgtcaa tcccg |
| Primer 6F | gccgaaagca gacctgatcc catatag |
| Primer 5 R | gttttctttctttccgctagtcaccag |
| Primer 6 R | gataatggctgacttaccagatcctg |
| Primer 7 F | gtcatagga gtcttcgggg taccagg |
| Primer 8 F | ccaggcaa gacctggtga ctagc |
| PRIMER 7 R | cgccattatcgatgcgtgctc |
| PRIMER 8 R | gtcaccagagagtgtcttccataccag |
| Primer 9 F | gg aagacactct ctggtgaccc g |
| Primer 10F | ggaaacttc aaggcaacta ttaagg |
| PRIMER 9 R | gcactgttggtaatggtgtatgcg |
| PRIMER 10R | ctaccaagtgttgccggtagac |
| Primer 11 F | cggagcggac tatacataca acc |
| Primer 12F | cctagtggtc ataaacatcc acac |
| PRIMER 11 R | gttagcctgtctttccctcctgag |
| PRIMER 12R | gatgaccacgtctgcatccgtcg |
| Primer 13 F | cgtagct atacctctcc tctc |
| Primer 14F | ccagtcactg aaccacctct ttac |
| PRIMER 13 R | ggtcgtatgtatcgccccg |
| PRIMER 14R | gcatccaggtctgacgggacgg |
| Primer 15 F | cagttt gatctaagcg ccgatggcga gac |
| Primer 16 F | gacgcccca gccctagaac cgg |
| PRIMER 15R | cggggtctctgccattaaatac |
| PRIMER 16 R | gtttacagcctctctttagtctctg |
| Primer 17 F | gtccacggcc aatagaagca gg |
| Primer 18 F | catccagaga ctaaagagag gc |
| PRIMER 17 R | gcgttcaatctcctaaccaattctctg |
| PRIMER 18 R | cagccgcctgtataacctgcacc |
| Primer 19 F | caaagcata cagaggaaag gcc |
| Primer 20F | ctga acccttggca acagcgtacc |
| PRIMER 19 R | cttcgtacctagagtacaccgc |
| PRIMER 20R | ctgattacttcatcagccagcgc |
| Primer 21 F | gcggcaggt gacgaacaag acgaag |
| Primer 22 F | ggcaacga acagggctaa tag |
| PRIMER 21 % | gtcgtacttagatgaccgcttgaag |
| PRIMER 22 R | gtgaacttcgaagcgtcggacttcatg |
| Primer 23 F | ccatcgata acgcggacct ggcc |
| Primer 24F | gaatgcgcgc agatacccgt gca |
| PRIMER 23 R | ggcatgtgattgtccatataacg |
| PRIMER 24R | cattgttccagtaatcgtgcac |
| Primer 25F | cggatgata gccatgattg gacc |
| Primer 26F | gcagagcggg ccgggctatt tgtaag |
| PRIMER 25R | cttcttatgcgtcacccactcttc |
| PRIMER 26R | cttgtacggctcattgttaccc |
| Primer 27 F | gaacc aaactatcaa gaagagtgg |
| Primer 28F | ggttaaccg tgccgactga ggg |
| PRIMER 27 R | gtaggcgccgccccacatgaatgg |
| PRIMER 28R | ggctcttgtccttacactctgctg |
| Primer 29F | gttatcccgt ctccgtacgt gaaatg |
| Primer 30F | cctgattaca gctgtaaggt cttcac |
| PRIMER 29R | cccaaagtctgaggaatgggtgc |
| PRIMER 30R | catcgagtgcactgcacacttgcc |
| Primer 31F | ggacatgtc gtgtgaggta tcag |
| Primer 32F | ggcgtagcca tcattaaata tgcag |
| PRIMER 31R | ctcttccgattgccaattatggtattc |
| PRIMER 32R | gcctacatctcaaagcgagttcgg |
| Primer 33 F | gtaacaaaa tataaaacta ataaaaatca |
| Primer 34 F | gtaggtac ttaagcttct taaaagcagc |
| PRIMER 33R | aatattaaaaacaaaataacatctcctacg |
| PRIMER 34R | cggagaattgtggaagagttcggtatgc |
| 3’RACE F | ccgaactct tccataattc tcc |
| T25G | TTTTTTTTTTTTTTTTTTTTTTTTTG |

**Supplementary Figure S1a**

ZIKV phylogeny, complete tree

Arrows indicate the ZIKV strain sequenced in the present study


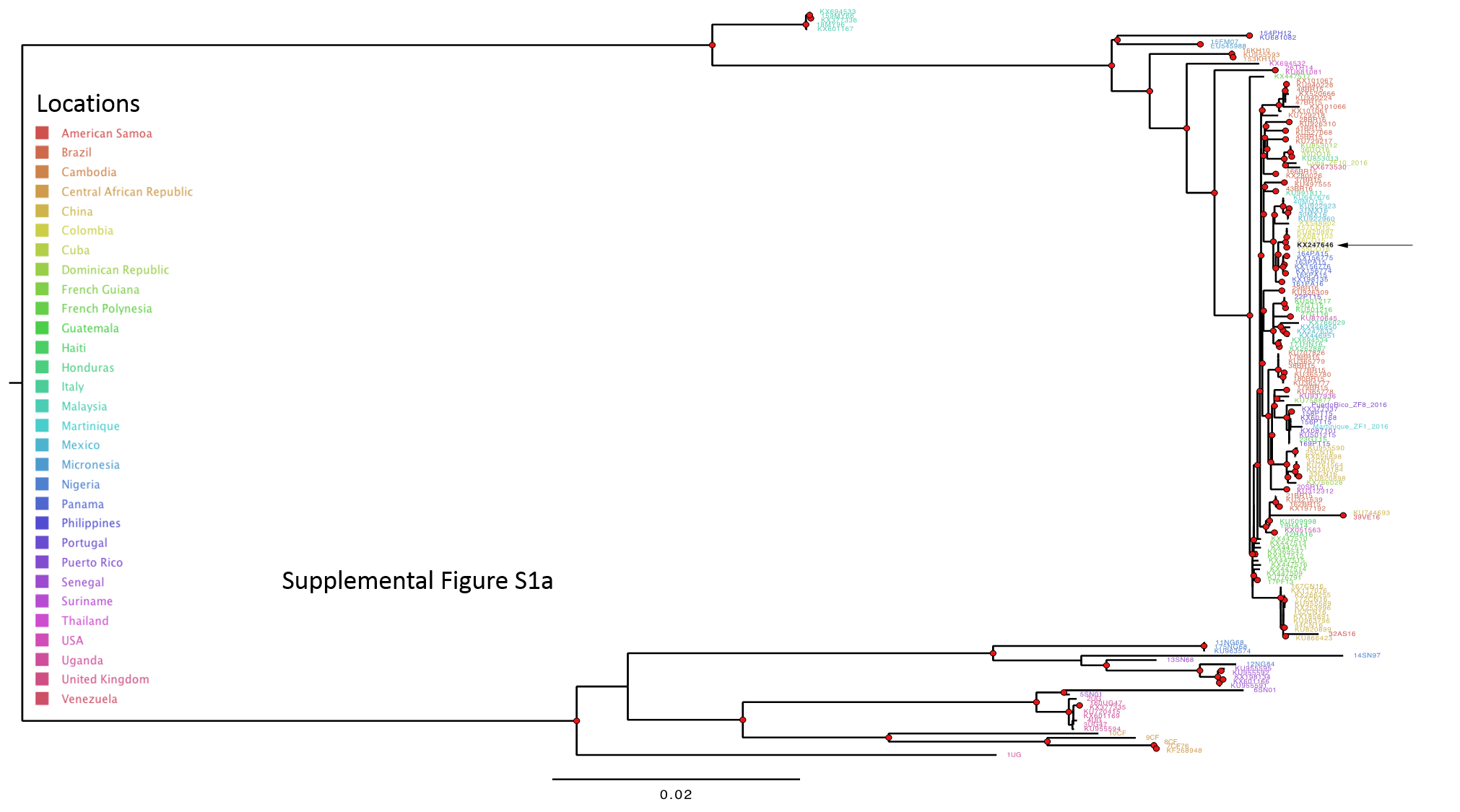


**Supplementary Figure S1b**

CHIKV phylogeny, complete tree

Arrows indicate the CHIKV strain sequenced in the present study


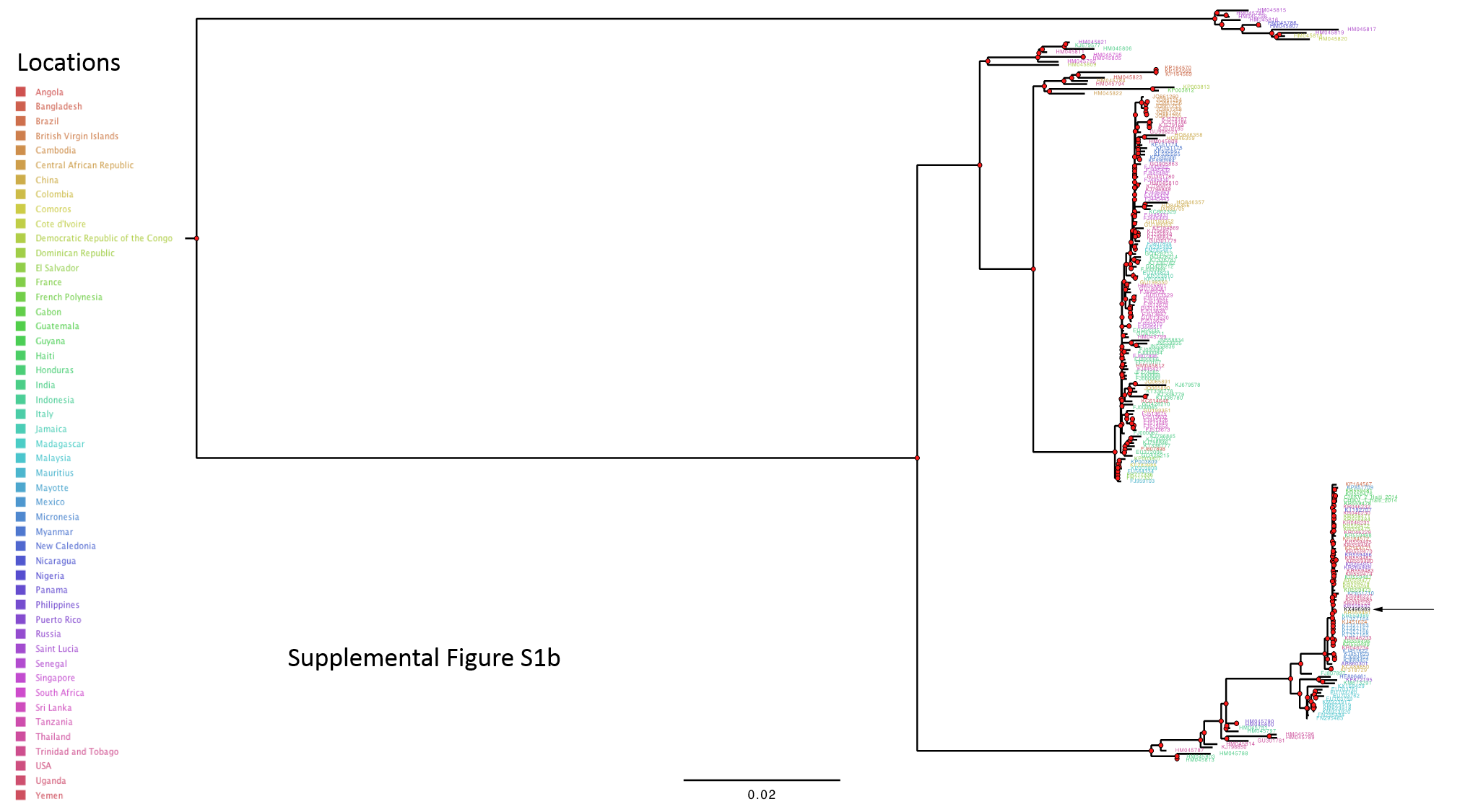

Supplement: Supplementary File 1 [file jmmcr-03-5072-s001.docx]
